# Editorial

**Published:** 1861-06

**Authors:** 


### Editorial.

---

Through a miscalculation, several articles, including our usual selections of medical intelligence, etc., already prepared, and some actually in type, have been crowded out.

We shall be pleased to have, from any of the Medical Staff of the Western Division of the Army, the records of their observations and experience, for publication in the Examiner, for the benefit and instruction of the profession.

The *American Medical Times*, in a recent number transfers thirty of our Illinois physicians, *en masse*, to Michigan, by making the Illinois Medical Examining Board an appointment of the Legislature of Michigan. Illinois is still in the Union, Messieurs Editors *American Medical Times*, and not a peninsular dependency.

Summer Schools.—The Summer Course of the Medical Department of Lind University is still in progress, and the students are now enjoying the advantage of cliniques in the Marine Hospital—making in all, eight cliniques per week, at the present time, and six didactic lectures.

We are informed that the Summer Course of the Rush Medical College has been discontinued, for the past two or three weeks, nor do we learn of any intention to resume until the usual winter term.

Contagiousness of Diphtheria.—The recent death of Dr. Horace W. Adams, of Boston, from diphtheria, contracted from a patient, reminds us to suggest to our readers the propriety of placing on record the details of cases of such contagion, falling under their observation. Foreign exchanges mention the death of M. Gendron, of Château du Loire, France, from a similar cause,—the 'diptheritic exudations having been received in his face and mouth, while performing the operation of tracheotomy on a patient under his care. Dr.

Frick, of Baltimore, also died, it may be remembered, in a similar manner, last year. The Berkshire *Medical Journal* regards an immediate thorough washing with some chlorinated solution, such as Labarraque's, or of diluted hydrochloric acid, as the means most likely to avert the risk of contagion, in case of receiving the false membrane on a mucous surface. The acidulated solution of chlorate of potash, after the formula and mode of use given on page 159 of the current volume of the EXAMINER, will no doubt, be found efficient in such cases.

OBITUARY—DR. LAWSON—DR. REESE.—The venerable head of the Medical Department of the United States Army, died suddenly of apoplexy, during the past month, at the advanced age of seventy-five. Surgeon-General LAWSON had held his position for over twenty years, and during his half century's experience of practice, had won for himself an enviable reputation as a successful physician, and an honorable gentleman and soldier. He died at Norfolk, whither he had gone for the benefit of his health.

——The well known editor of the *American Medical Gazette*, DAVID MEREDITH REESE, M. D., LL.D., died at his residence in New York city, on the morning of the 13th ult., of cardiac disease. Dr. Reese's name has been quite prominently before the profession in the forty years of an active career, during which he has filled chairs in the Washington University of Baltimore, in the Albany Medical College, N. Y., in the Castleton Medical College, Vt.; was for several years Resident Physician to Bellevue Hospital; Vice President of the American Medical Association; edited, and was author of several works of a professional and general character; and at the time of his death, held a professorship in the New York Medical College, and during the last session, delivered a full course of lectures on his branch—the Practice of Medicine. The *Am. Med. Times* says his most useful papers were his reports to the American Medical Association, the last of which, on *Medical Education*, it characterizes as replete with mature and well-digested views on this all-important subject.

SCURVY.—The remarks of Prof. Andrews on this subject (see page 259) derive additional interest from the fact that this disease is on the increase in the mercantile marine, and that in the United States Army, 2,803 cases of scurvy were reported as occurring, in the five years ending December, 1859, in an average number of less than fourteen thousand men.

### Special Notices.

SUMMER TERM AT THE LIND.—*One recitation and one familiar explanatory lecture daily until the first Monday in October, on the following branches:—Materia Medica and Prac. Med.—Prof. J. H. Hollister. Anatomy and Surgery—Prof. R. N. Isham. Chemistry—Prof. F. Mahla. Obstetrics and Diseases of Women—Prof. W. H. Byford. Physiology and Pathology—Prof. H. A. Johnson. Clinical Surgery—Prof. E. Andrews. Clinical Medicine—Prof. N. S. Davis.*

*Cliniques at Mercy Hospital, from* 9 *to* 10 *A. M., in the Medical Wards, service of* Prof. DAVIS, *on Tuesdays and Fridays; in the Surgical Wards, service of* Prof. ANDREWS, *on Mondays and Thursdays. At Marine Hospital, from* 9 *to* 10 *A. M., service of* Prof. ISHAM, *on Wednesdays and Saturdays.*

*At Chicago Dispensary, from* 2 *to* 3 *P. M., Medical and Surgical service,* Profs. ANDREWS *and* BYFORD, *on Wednesdays; Diseases of Women and Children, service* Prof. BYFORD, *on Saturdays.*

*Lectures on Military Surgery, by* Prof. ANDREWS, *in the Amphitheatre of the College, on Wednesday and Saturday afternoons, from* 5 *to* 6.

Prof. BRAINARD *lectures on Military Surgery, in the lower lecture-room of the Rush Medical College, on Tuesdays and Fridays, from* 5 *to* 6 *P. M.*

PHYSICIANS, *willing to propagate Vaccine symph, for the use of the volunteer forces of this State, can obtain a few charges by applying at the office of the* EXAMINER, *with the understanding that the increase is to be devoted to this purpose.*

